# Confirmatory psychometric evaluations of the Impact of Weight on Quality of Life–Lite Clinical Trials Version (IWQOL‐Lite‐CT)

**DOI:** 10.1111/cob.12477

**Published:** 2021-07-22

**Authors:** Ronette L. Kolotkin, Valerie S. L. Williams, Lisa von Huth Smith, Henrik Hjorth Meincke, Shanshan Qin, Nicole Williams, Sheri E. Fehnel

**Affiliations:** ^1^ Quality of Life Consulting Durham North Carolina USA; ^2^ Department of Family Medicine and Community Health Duke University School of Medicine Durham North Carolina USA; ^3^ Faculty of Health and Social Sciences Western Norway University of Applied Sciences Førde Norway; ^4^ Centre of Health Research Førde Hospital Trust Førde Norway; ^5^ Morbid Obesity Centre Vestfold Hospital Trust Tønsberg Norway; ^6^ Department of Patient Reported Outcomes RTI Health Solutions Research Triangle Park North Carolina USA; ^7^ Novo Nordisk A/S Søborg Denmark

**Keywords:** clinical trials, functioning, health‐related quality of life, obesity, overweight, validation

## Abstract

The Impact of Weight on Quality of Life–Lite Clinical Trials Version (IWQOL‐Lite‐CT) was developed to assess weight‐related physical and psychosocial functioning in the context of clinical trials. Data from two pivotal trials of once‐weekly subcutaneous semaglutide for the purpose of weight management (NCT03548935 and NCT03552757) were analysed to confirm the structure, reliability, validity, and responsiveness of the IWQOL‐Lite‐CT and evaluate the magnitude of meaningful within‐patient change in patients with overweight or obesity, with and without type 2 diabetes. Factor analyses and inter‐item correlations confirmed the IWQOL‐Lite‐CT structure and scoring algorithm. Each composite score (physical, physical function, psychosocial, and total) demonstrated excellent internal consistency (Cronbach's alphas ≥ 0.82) and test–retest reliability (intraclass correlation coefficients ≥ 0.85) in both trials. Patterns of cross‐sectional and longitudinal construct validity correlations were generally consistent with hypotheses. Each of the IWQOL‐Lite‐CT composites was able to discriminate between known groups. Effect sizes and paired *t* tests comparing IWQOL‐Lite‐CT scores at baseline and Week 68 were statistically significant for all composites in both trials (*P* < 0.0001), providing strong support for the ability to detect change. Results of anchor‐based analyses supported responder thresholds ranging from 13.5 to 16.6 across composite scores. The IWQOL‐Lite‐CT, a comprehensive assessment of weight‐related functioning from the patient perspective, is appropriate for use in clinical trials evaluating the efficacy of new treatments for weight management.

**What is already known about this subject?**

- Among individuals with obesity, treatment has the potential to improve aspects of functioning and health‐related quality of life (HRQOL); thus, these concepts are important outcomes in evaluations of weight‐management interventions.
- While changes in patient‐reported outcomes (PROs), including patient functioning, are commonly key outcomes in weight management trials, they are rarely mentioned in product labelling, particularly in the United States (US).
- The Impact of Weight on Quality of Life–Lite Clinical Trials Version (IWQOL‐Lite‐CT) is a PRO measure of weight‐related functioning, developed in accordance with recommendations published by the US Food and Drug Administration (FDA) for measures used to support labelling, based on information gleaned from the obesity literature, qualitative research conducted with patients, clinical experts, and consultation with the FDA.

**What this study adds?**

- Results of the psychometric analyses presented here confirm the reliability, validity, and responsiveness and provide estimates of meaningful within‐patient change on the final 20‐item version of the IWQOL‐Lite‐CT, further contributing to the body of evidence supporting the IWQOL‐Lite‐CT.
- The IWQOL‐Lite‐CT is appropriate for assessing weight‐related physical and psychosocial functioning in populations commonly targeted for weight management clinical trials.

## 1 INTRODUCTION

Obesity is a chronic disease with adverse health, social, psychological, and economic consequences.^1^, ^2^ In their patient‐centred disease model, for example, Fastenau and colleagues^3^ describe negative impacts of obesity on physical functioning, social/leisure functioning, emotional functioning, psychological functioning, sexual functioning, and work productivity, as well as comorbid conditions and other aspects of the patient's life. Among individuals with obesity, weight reduction is commonly accompanied by improvements in health‐related quality of life (HRQOL), with subsequent changes in HRQOL generally matching long‐term patterns of weight loss, gain, and stability.^4^ Because treatment has the potential to improve various aspects of functioning and HRQOL among patients with obesity, these concepts are important outcomes in evaluations of weight‐loss and weight‐management interventions, including behavioural, psychological, surgical, and pharmaceutical treatments.^4^, ^5^, ^6^, ^7^, ^8^, ^9^, ^10^

In their review of patient‐reported outcome (PRO) measures used to assess HRQOL in the context of obesity, Wadden and Phelan (2002) describe several generic measures, including the Short Form Health Survey (SF‐36), the Nottingham Health Profile (NHP), and the Sickness Impact Profile (SIP), which have demonstrated the ability to capture improvements associated with weight loss.^11^ While recommending use of the SF‐36 among the generic measures of HRQOL, the authors note that by capturing impacts most salient to patients, disease‐specific measures tend to be more sensitive to change than generic measures. At least four obesity‐specific measures of HRQOL are available. The 31‐item Impact of Weight on Quality of Life–Lite (IWQOL‐Lite) was developed to evaluate the impact of obesity on HRQOL and functioning in individuals with obesity in a variety of settings.^12^ The 17‐item Obesity and Weight‐Loss Quality‐of‐Life (OWLQOL) was developed to evaluate HRQOL in individuals with obesity or who are trying to lose weight,^13^ whereas the 6‐item Moorehead‐Ardelt Quality of Life is a measure of HRQOL specifically developed for a postoperative population.^14^ In addition, a 140‐item battery constructed for use in the Swedish Obesity Study (SOS), which evaluated surgically treated individuals with severe obesity compared with a conventionally treated control group, included an 8‐item obesity‐related problems scale used to measure the impact of obesity on psychosocial functioning.^4^ In a systematic review of research examining the effects of obesity and weight loss on HRQOL (based on the results of 12 previously published reviews), Kolotkin and Andersen^15^ found that the IWQOL‐Lite^12^ was used more commonly than any other obesity‐specific measure and consistently demonstrated an association between obesity and reduced HRQOL.

Although the IWQOL‐Lite has also performed well in numerous evaluations of pharmacological,^16^, ^17^ surgical,^18^, ^19^ and dietary^20^ interventions for obesity, the content of this questionnaire was largely based on the input of individuals receiving residential treatment for obesity.^12^ As such, this measure may not be ideal for demonstrating treatment benefit among populations participating in clinical trials of pharmacological interventions, which typically include individuals with lesser degrees of obesity and fewer comorbid conditions than those who seek such intensive treatment. Furthermore, this measure was developed prior to the publication of the US Food and Drug Administration's (FDA's) Guidance for Industry Patient‐Reported Outcome Measures: Use in Medical Product Development to Support Labeling Claims (PRO Guidance),^21^ which may also limit its acceptance by the FDA and possibly other regulatory authorities to support product labelling claims.

A variant of the IWQOL‐Lite, the Impact of Weight on Quality of Life–Lite Clinical Trials Version (IWQOL‐Lite‐CT), was recently developed specifically for use in clinical trials.^22^, ^23^ While both the IWQOL‐Lite and IWQOL‐Lite‐CT assess HRQOL‐related concerns of particular relevance to patients with overweight and obesity, the IWQOL‐Lite‐CT focuses on aspects of physical and psychosocial functioning likely to change with modest (10%) weight loss among populations most commonly targeted for participation in clinical trials of pharmaceutical products for weight loss and weight management. Importantly, this measure was developed in accordance with FDA guidance documents pertaining to PRO measures used to support product labeling^21^, ^24^ based on information gleaned from the obesity literature, qualitative research conducted with patients, clinical experts, and consultation with the FDA.^22^ Development also aligned with current standards described by professional organizations such as the International Society for Quality of Life Research (ISOQOL).^25^ Furthermore, developmental versions of the IWQOL‐Lite‐CT have been shown to be reliable, valid, and responsive measures of weight‐related functioning in the populations commonly targeted for clinical trials of new weight‐management medications.^23^

The objectives of this study were to supplement the evidence supporting the IWQOL‐Lite‐CT by confirming the reliability, validity, and responsiveness and providing estimates of meaningful within‐patient change on the final 20‐item version of this measure, using data from pharmacological trials for weight management.

## 2 MATERIALS AND METHODS

### 2.1 Study population

Confirmatory psychometric evaluations of the IWQOL‐Lite‐CT were conducted with the use of data from two multinational phase 3a clinical trials of semaglutide for weight management, STEP 1 (NCT03548935) and STEP 2 (NCT03552757).^26^, ^27^, ^28^ Both trials were designed to compare the efficacy and safety of once‐weekly semaglutide (2.4 mg administered subcutaneously for 68 weeks) with placebo, as an adjunct to a reduced calorie diet and increased physical activity. STEP 1 included nondiabetic patients with overweight (body mass index [BMI] ≥ 27 kg/m^2^ to <30 kg/m^2^) in the presence of at least one weight‐related comorbidity or obesity (BMI ≥ 30.0 kg/m^2^). STEP 2 included patients with type 2 diabetes (T2D) in addition to overweight or obesity (BMI ≥ 27.0 kg/m^2^). Psychometric analyses were conducted using all randomized patients in the full analysis set who completed the baseline IWQOL‐Lite‐CT assessment (*n* = 1945 in STEP 1; *n* = 1186 in STEP 2).

### 2.2 Measures

The IWQOL‐Lite‐CT is a 20‐item patient‐reported outcome (PRO) measure designed to assess the impact of changes in weight on patients' physical and psychosocial functioning. Each item employs a 5‐point graded response scale (never, rarely, sometimes, usually, always; or not at all true, a little true, moderately true, mostly true, completely true). In addition to yielding a Total score, the final 20‐item IWQOL‐Lite‐CT includes two primary domains: Physical (7 items) and Psychosocial (13 items). Based on feedback from the FDA and to facilitate labelling in the United States (US), a 5‐item subset of the Physical domain, the Physical Function composite, has also been evaluated and supported.^23^

**FIGURE 1:**
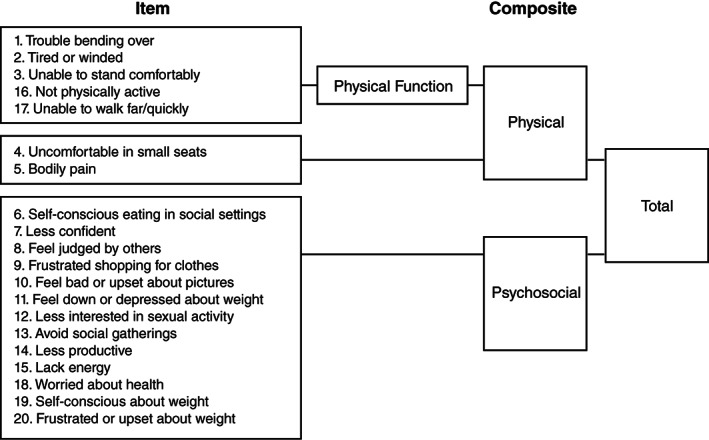
IWQOL‐Lite‐CT conceptual framework. IWQOL‐Lite‐CT, Impact of Weight on Quality of Life–Lite Clinical Trials Version

A conceptual framework for the IWQOL‐Lite‐CT is depicted in Figure 1.

The IWQOL‐Lite‐CT is generally scored according to the rules of the IWQOL‐Lite^29^ to yield composite scores and a Total score ranging from 0 to 100, with higher scores reflecting better levels of functioning.

In addition to weight, BMI, and the IWQOL‐Lite‐CT, the psychometric evaluation utilized data from the Short Form Health Survey–36, Version 2 Acute (SF‐36v2), Patient Global Impression of Change (PGI‐C) items pertaining to physical functioning and mental health, and Patient Global Impression of Status (PGI‐S) items pertaining to physical functioning and mental health. Table 1 summarizes these measures in further detail.

**TABLE 1:** Outcome measures used in the analysis

Measure	Recall period and response scale	Scoring
**Patient global impressions of status** PGI‐S PF: How would you rate your physical functioning (mobility and ability to do physical activities) at your current weight? PGI‐S MH: How would you rate how you feel mentally (emotions, self‐confidence) at your current weight?	Current 5‐point verbal response scale ranging from “poor” to “excellent”	Item scores range from 1 to 5 Higher scores reflect better outcomes
**Patient global impressions of change** PGI‐C PF: How would you rate your physical functioning (mobility and ability to do physical activities) at your current weight as compared to the beginning of the study? PGI‐C MH: How would you rate how you feel (emotions, self‐confidence) at your current weight as compared to the beginning of the study?	Change since baseline 7‐point verbal response scale ranging from “much better” to “much worse”	Item scores range from 1 to 7 Higher scores reflect worse outcomes
**Short Form Health Survey–36 36 items** Two summary scores: ▪ Physical component summary ▪ Mental component summary Eight subscales: ▪ Physical functioning ▪ Role‐physical ▪ Bodily pain ▪ Social functioning ▪ General mental health ▪ Role‐emotional ▪ Vitality ▪ General health perceptions	1 week Various ordinal item response scales	Component and subscale scores converted to 2009 US norm‐based scores (mean = 50, SD = 10) Higher scores indicate better functioning

Abbreviations: MH, mental health; PF, physical functioning; PGI‐C, patient global impression of change; PGI‐S, patient global impression of status.

### 2.3 Analytic methods

Each of the following analyses were conducted separately with the use of data from STEP 1 and STEP 2 to evaluate and support the measurement properties of the IWQOL‐Lite‐CT for use in patients with overweight and obesity, with and without T2D.

- Standard descriptive statistics were computed to characterize the sample, and item‐level response frequency distributions were examined for floor and ceiling effects for each IWQOL‐Lite‐CT item.
- To confirm the two‐domain structure (physical and psychosocial composites) supported by the previous psychometric evaluations and qualitative research, longitudinal confirmatory factor analyses (CFAs) were conducted with the use of baseline, Week 20, and Week 68 data.
- Two models were tested with the use of data from each clinical trial, yielding a total of four models. Scale invariance was imposed within each model such that the unstandardized factor loadings and intercepts were constrained to equality across time points, and the residuals of each item were also allowed to be correlated across time points. In the first 2‐factor model tested within each trial, the IWQOL‐Lite‐CT items were allowed to load only on the factor with which they were grouped for scoring purposes. The second 2‐factor model was based on the residual correlations and the modification indices from the first CFA model as well as the results of previous IWQOL‐Lite‐CT factor analyses. Goodness‐of‐fit indices were also evaluated.
- Cronbach's^30^ coefficient alpha was computed to evaluate the internal consistency of the IWQOL‐Lite‐CT composites (total, physical, physical function, and psychosocial) at four time points: baseline, Week 16, Week 20, and Week 68. The approximate range of optimal alphas is between 0.70 and 0.90, indicating a set of strongly related items capable of supporting a composite score but not redundant.
- To evaluate test–retest reliability, intraclass correlation coefficients (ICCs) for the IWQOL‐Lite‐CT composite scores using subsets of stable patients defined by body weight and PGI‐S ratings. “Test” and “retest” data were IWQOL‐Lite‐CT scores obtained at Week 16 and Week 20, respectively, from patients with 5% or less change in body weight and who rated themselves the same on the corresponding PGI‐S items at both time points.
- To evaluate cross‐sectional construct validity, correlations were computed between IWQOL‐Lite‐CT composite scores and scores on the SF‐36v2 (subscale, physical component summary [PCS], and mental component summary [MCS] scores) and PGI‐S items. To evaluate longitudinal construct validity, correlations were computed between changes in IWQOL‐Lite‐CT composite scores and changes in the same patient‐reported measures from baseline to Week 68, as well as PGI‐C item scores at Week 68.
- It was hypothesized that the IWQOL‐Lite‐CT Physical and Physical Function scores would be at least moderately correlated (|*r*| ≥ 0.30) with the SF‐36v2 PCS and the SF‐36v2 Physical Functioning, Role‐Physical, and Vitality subscale scores, as well as PGI‐S Physical Functioning (PF) scores. Similarly, it was hypothesized that the IWQOL‐Lite‐CT Psychosocial composite would be at least moderately correlated with the SF‐36v2 MCS and the SF‐36v2 mental health (MH) and vitality subscale scores, as well as PGI‐S MH scores. At least moderate correlations among change scores based on these measures were also hypothesized. At least moderate correlations were also hypothesized between changes in the IWQOL‐Lite‐CT Physical and Physical Function composite scores with the PGI‐C PF item and between change in the IWQOL‐Lite‐CT psychosocial composite score and the PGI‐C MH item.
- To evaluate discriminating ability, known‐groups analyses of variance were used to examine mean differences in IWQOL‐Lite‐CT composite scores between patients classified into subgroups based on BMI and percentage of weight loss. It was hypothesized that better IWQOL‐Lite‐CT scores would be observed in patients with lower BMIs and greater weight loss.
- To evaluate the responsiveness of the IWQOL‐Lite‐CT, effect sizes, standardized response means, and paired *t* tests compared the differences in each composite score between baseline and Week 68. Effect size estimates of approximately 0.20 are considered small, those of approximately 0.50 are moderate, and those greater than approximately 0.80 are large.^31^
- Finally, anchor‐based analyses, conducted in line with FDA guidance,^21^ were conducted to estimate responder thresholds (i.e., thresholds for clinically meaningful within‐patient improvements) for each composite score based on changes in PGI‐S item scores from baseline to Week 68 and responses to PGI‐C items at Week 68. Specifically, thresholds for the IWQOL‐Lite‐CT composite scores were computed as the average changes from baseline to Week 68 among patients who reported a 1‐point improvement on the corresponding PGI‐S item and patients who reported they were “moderately better” on the corresponding PGI‐C item (i.e., PGI‐S PF and PGI‐C PF for the Physical and Physical Functioning composites and PGI‐S MH and PGI‐C MH for the Psychosocial composite). Thresholds for the IWQOL‐Lite‐CT Total score were computed as the average change from baseline to Week 68 of patients who reported a 1‐point improvement on both the PGI‐S PF and the PGI‐S MH, as well as patients who reported they were “moderately better” on both the PGI‐C PF and the PGI‐C MH. For the purpose of responder analyses using the trial data, a 1‐point improvement in PGI‐S score from baseline to Week 68 within STEP 1 was identified as the primary anchor. Supportive, distribution‐based methods (including half standard deviation [SD] and standard error of the mean) described in FDA guidance documents^21^, ^24^ were also applied to provide support for the anchor‐based responder thresholds.

## 3 RESULTS

### 3.1 Participant characteristics

The analysis population included trial participants who completed a baseline IWQOL‐Lite‐CT assessment (*n* = 1945 in STEP 1; *n* = 1186 in STEP 2). Table 2 presents key baseline characteristics of the analysis population. Patients participating in STEP 1 ranged in age from 18 to 86 years, with a mean age of 46.5 years. The majority of patients were female (*n* = 1440, 74.0%) and the majority were white (*n* = 1457, 77.1%). The baseline BMIs of patients ranged from 26.5 kg/m^2^ to 83.0 kg/m^2^, with a mean of 37.9 (SD = 6.66). Patients participating in STEP 2 ranged in age from 19 to 84 years, with a mean age of 55.3 years. The majority of patients were white (*n* = 733, 61.8%), and the sex composition of the sample was roughly evenly split, with 604 (50.9%) females and 582 (49.1%) males. The mean BMI at baseline was 35.7 kg/m^2^ (SD = 6.30), ranging from 26.5 kg/m^2^ to 66.2 kg/m^2^.

**TABLE 2:** Patient characteristics at baseline^a^

Patient characteristic	Step 1	Step 2
	Overall (*N* = 1945)	Overall (*N* = 1186)
**Age, mean (SD), y**	46.5 (12.71)	55.3 (10.58)
Median, minimum‐maximum	47.0, 18.0–86.0	56.0, 19.0–84.0
**Sex, *n* (%)**		
Male	505 (26.0)	582 (49.1)
Female	1440 (74.0)	604 (50.9)
**Height, mean (SD), *m***	1.7 (0.09)	1.7 (0.10)
Median, minimum‐maximum	1.7, 1.4–2.0	1.7, 1.3–2.0
**Weight, mean (SD), kg**	105.3 (21.84)	99.7 (21.45)
Median, minimum‐maximum	101.9, 61.8–245.6	97.1, 54.4–199.2
**Body mass index, mean (SD)**	37.9 (6.66)	35.7 (6.30)
Median, minimum‐maximum	36.6, 26.5–83.0	34.3, 26.5–66.2
**Race, *n* (%)**		
Asian	260 (13.8)	315 (26.6)
Black or African American	111 (5.9)	96 (8.1)
White	1457 (77.1)	733 (61.8)
Native Hawaiian or other Pacific Islander	2 (0.1)	1 (0.1)
American Indian or Alaska Native	27 (1.4)	6 (0.5)
Other	33 (1.7)	35 (3.0)
**Ethnicity, *n* (%)**		
Hispanic or Latino	229 (12.1)	150 (12.6)

^a^ The analysis population included only STEP 1 and STEP 2 participants who completed a baseline IWQOL‐Lite‐CT assessment.

### 3.2 IWQOL‐Lite‐CT structure

Table S1 and Table S2 (Supporting Information) present the CFA results based on the STEP 1 and STEP 2 data, respectively. In STEP 1, the first 2‐factor CFA model yielded a satisfactory root mean square error of approximation (RMSEA) of 0.059 and an acceptable standardized root mean square residual (SRMR) of 0.063, but the comparative fit index (CFI) and Tucker‐Lewis Index (TLI) values (0.936 and 0.938, respectively) were somewhat lower than the conventional cutoff of 0.95. For the Physical factor, the loadings were very similar in size, ranging from 0.69 to 0.81. For the Psychosocial factor, the loadings ranged from 0.58 to 0.87.

The second CFA model conducted with the use of data from STEP 1 exhibited satisfactory goodness‐of‐fit, with a somewhat better RMSEA and SRMR (both < 0.06) and better CFI and TLI values (both = 0.95). For the Physical factor, the loadings ranged from 0.69 to 0.79. For the Psychosocial factor, the loadings ranged from 0.57 to 0.88. As shown in Table S2, the CFA results based on the data from STEP 2 were very consistent with those based on the STEP 1 data, further confirming and supporting the structure and scoring of the IWQOL‐Lite‐CT.

### 3.3 Reliability

As shown in Table 3, internal consistency results were strong for all composite scores at all‐time points in both STEP 1 and STEP 2 (alpha ≥ 0.82), further supporting the IWQOL‐Lite‐CT scoring algorithm.

**TABLE 3:** Summary of key measurement properties of IWQOL‐Lite‐CT composites

Measurement property	STEP 1/ STEP 2 results of IWQOL‐Lite‐CT scores			
	Total	Physical	Physical Function	Psychosocial
**Distribution: baseline, *n* = 1945/1186; Week 68, *n* = 1761/1111**				
Mean (SD) at baseline	63.49 (21.08)/73.50 (19.60)	64.35 (23.14)/68.63 (23.03)	64.95 (24.13)/69.16 (23.96)	63.02 (22.91)/76.13 (20.39)
Mean (SD) at Week 68	76.84 (19.66)/81.64 (17.31)	75.78 (22.13)/76.70 (22.23)	77.37 (22.76)/77.86 (22.98)	77.41 (20.69)/84.30 (16.91)
Patients with lowest score (floor effect) at baseline, %	0.0/0.0	0.5/0.6	0.8/0.7	0.2/0.2
Patients with highest score (ceiling effect) at baseline, %	1.3/4.0	4.0/7.1	6.8/9.5	2.6/8.0
**Internal consistency**				
Cronbach's alpha at baseline	0.94/0.94	0.86/0.87	0.82/0.83	0.94/0.93
Cronbach's alpha at Week 68	0.95/0.94	0.89/0.90	0.87/0.88	0.94/0.93
**Test–retest reliability: Week 16 to Week 20**				
ICC (*n*)	0.92 (670)/0.90 (416)	0.88 (1051)/0.87 (633)	0.86 (1051)/0.85 (633)	0.92 (996)/0.87 (606)
**Cross‐sectional construct validity correlations at baseline (*n* = 1945/1186)**				
SF‐36v2 MCS	0.28/0.21	0.10/0.11	0.10/0.09	0.33/0.25
SF‐36v2 PCS	0.58/0.59	0.73/0.71	0.72/0.71	0.42/0.43
SF‐36v2 PF	0.52/0.48	0.67/0.63	0.66/0.64	0.37/0.33
SF‐36v2 RP	0.46/0.46	0.55/0.55	0.56/0.56	0.36/0.35
SF‐36v2 BP	0.49/0.50	0.61/0.59	0.58/0.56	0.36/0.38
SF‐36v2 GH	0.49/0.51	0.49/0.50	0.47/0.49	0.43/0.45
SF‐36v2 VT	0.61/0.56	0.54/0.55	0.52/0.54	0.57/0.49
SF‐36v2 SF	0.40/0.34	0.36/0.32	0.35/0.31	0.37/0.31
SF‐36v2 RE	0.25/0.24	0.19/0.25	0.20/0.25	0.24/0.21
SF‐36v2 MH	0.36/0.30	0.27/0.25	0.26/0.24	0.37/0.29
PGI‐S PF	0.55/0.57	0.61/0.58	0.61/0.58	0.45/0.49
PGI‐S MH	0.56/0.47	0.37/0.35	0.37/0.33	0.59/0.49
**Longitudinal correlations of changes from baseline to Week 68, *n* = 1760 to 1761/1110 to 1111**				
SF‐36v2 MCS	0.27/0.23	0.20/0.21	0.19/0.20	0.28/0.20
SF‐36v2 PCS	0.50/0.46	0.60/0.53	0.57/0.52	0.39/0.34
SF‐36v2 PF	0.48/0.41	0.56/0.49	0.55/0.49	0.38/0.28
SF‐36v2 RP	0.40/0.38	0.46/0.42	0.46/0.42	0.32/0.28
SF‐36v2 BP	0.37/0.34	0.44/0.40	0.40/0.36	0.29/0.24
SF‐36v2 GH	0.47/0.41	0.45/0.38	0.42/0.37	0.43/0.36
SF‐36v2 VT	0.48/0.39	0.45/0.39	0.44/0.38	0.43/0.32
SF‐36v2 SF	0.32/0.27	0.33/0.27	0.32/0.26	0.28/0.23
SF‐36v2 RE	0.25/0.24	0.22/0.25	0.22/0.24	0.23/0.19
SF‐36v2 MH	0.34/0.27	0.28/0.29	0.27/0.27	0.33/0.21
PGI‐S PF	0.51/0.43	0.47/0.40	0.47/0.40	0.46/0.37
PGI‐S MH	0.49/0.42	0.36/0.32	0.36/0.31	0.50/0.41
PGI‐C PF	−0.47/–0.34	−0.41/–0.28	−0.39/–0.27	−0.45/–0.32
PGI‐C MH	−0.42/–0.32	−0.34/–0.26	−0.32/–0.25	−0.40/–0.31
**Known‐groups validity at Week 68: mean (SD) and *P* value**				
By BMI classes				
<35 kg/m^2^ (*n* = 1136/746)	82.1 (15.80)/85.2 (14.45)	81.3 (18.60)/81.0 (19.47)	82.9 (19.25)/82.1 (20.22)	82.5 (16.89)/87.5 (14.05)
>40 kg/m^2^ (*n* = 310/151)	63.8 (23.52)/70.3 (21.84)	61.1 (25.69)/62.9 (27.07)	62.6 (26.42)/63.9 (27.86)	65.3 (24.53)/74.2 (21.21)
*P* value	<0.0001/<0.0001	<0.0001/<0.0001	<0.0001/<0.0001	<0.0001/<0.0001
By weight change				
Loss ≥ 5% (*n* = 1215/583)	80.8 (16.95)/83.2 (16.54)	79.5 (19.90)/78.7 (21.13)	81.5 (20.24)/80.2 (21.69)	81.5 (17.82)/85.5 (16.13)
Gain > 0% (*n* = 257/156)	65.3 (22.85)/76.4 (19.65)	64.2 (24.90)/71.0 (24.66)	64.6 (25.81)/71.4 (25.38)	65.9 (24.46)/79.3 (19.80)
*P* value	<0.0001/<0.0001	<0.0001/<0.0001	<0.0001/<0.0001	<0.0001/<0.0001
**Ability to detect change**				
Mean change (SD)	13.02 (18.1)/7.86 (15.5)	11.17 (20.3)/7.70 (19.1)	12.18 (21.9)/8.29 (20.2)	14.01 (19.4)/7.95 (16.4)
Paired *t* test (*P* value)	−30.14 (<0.0001)/−16.87 (<0.0001)	−23.07 (<0.0001)/−13.43 (<0.0001)	−23.36 (<0.0001)/−13.68 (<0.0001)	−30.36 (<0.0001)/−16.19 (<0.0001)
ESE (SD_Baseline_)	0.62 (21.1)/0.40 (19.6)	0.48 (23.1)/0.33 (23.0)	0.50 (24.1)/0.35 (24.0)	0.61 (22.9)/0.39 (20.4)
SRM (SD_Change_)	0.72 (18.1)/0.51 (15.5)	0.55 (20.3)/0.40 (19.1)	0.56 (21.9)/0.41 (20.2)	0.72 (19.4)/0.49 (16.4)

Abbreviations: BMI, body mass index; BP, bodily pain; ESE, effect size estimate; GH, general health; ICC, intraclass correlation coefficient; IWQOL‐Lite‐CT, Impact of Weight on Quality of Life–Lite Clinical Trials Version; MCS, mental component summary; MH, mental health; PCS, physical component summary; PF, physical functioning; PGI‐S, patient global impression of status; RE, role‐emotional; RP, role‐Physical; SF, social functioning; SF‐36v2, Short Form Health Survey–36, Version 2 Acute; SRM, standardized response mean; VT, vitality.

Additionally, substantial test–retest agreement was observed among stable subjects, with ICCs ≥ 0.85 for all composite scores in both studies (Table 3). Specific to the Physical Function composite, ICCs were 0.86 in STEP 1 and 0.85 in STEP 2.

### 3.4 Construct validity

Observed patterns and magnitudes of construct validity correlations (both cross‐sectional and longitudinal) were generally consistent with hypotheses. In cross‐sectional analyses (Table 3), for example, IWQOL‐Lite‐CT Physical and Physical Function composite scores correlated strongly with PGI‐S PF and SF‐36v2 PCS scores, as well as SF‐36v2 Physical Functioning, Role‐Physical, and Vitality subscale scores at all three time points in both STEP 1 and STEP 2 (*r* = 0.52 to 0.75 across measures). In addition, IWQOL‐Lite‐CT Psychosocial scores correlated strongly with PGI‐S MH scores (*r* = 0.59 to 0.66), and correlated moderately with scores on the SF‐36v2 MCS, as well as the SF‐36v2 MH and Social Functioning subscales at all three time points in STEP 1 (*r* = 0.33 to 0.47 across measures). Correlations between IWQOL‐Lite‐CT psychosocial scores and scores on these other measures were similar but slightly lower in STEP 2, a trial in which smaller changes in weight and SF‐36v2 scores were observed.

In longitudinal analyses (Table 3), changes in IWQOL‐Lite‐CT Physical and Physical Function scores were moderately to strongly correlated with changes in scores on the PGI‐S Physical Function and the SF‐36v2 PCS, as well as the SF‐36v2 Physical Functioning, Role‐Physical, and Vitality subscales in both STEP 1 and STEP 2 (*r* = 0.38 to 0.60 across measures). While changes in IWQOL‐Lite‐CT Physical and Physical Function scores also correlated moderately with responses to the PGI‐C Physical Function in STEP 1 (*r* = −0.41 and −0.39, respectively), these relationships were slightly weaker in STEP 2 (*r* = −0.28 and −0.27, respectively). Similarly, changes in IWQOL‐Lite‐CT Psychosocial composite scores were moderately to strongly correlated with changes in scores on the PGI‐S MH in STEP 1 and STEP 2 (*r* = 0.50 and 0.41, respectively) and moderately correlated with responses to the PGI‐C MH in STEP 1 and STEP 2 (*r* = −0.40 and −0.31, respectively). In both STEP 1 and STEP 2, correlations were small to moderate in size between changes in IWQOL‐Lite‐CT Psychosocial composite scores and changes in SF‐36v2 MCS scores (*r* = 0.28 and 0.20, respectively), as well as changes in SF‐36v2 MH (*r* = 0.33 and 0.21, respectively) and Social Functioning (*r* = 0.28 and 0.23, respectively) subscale scores.

### 3.5 Discriminating ability

Known‐groups analyses of variance confirmed the ability of the IWQOL‐Lite‐CT composite scores to discriminate between groups based on current BMI and weight change in STEP 1 and STEP 2. Specifically, these analyses demonstrated that patients with BMIs < 35 kg/m^2^ had significantly higher IWQOL‐Lite‐CT scores compared to patients with BMIs > 40 kg/m^2^ (*P* < 0.0001 for all composites in both trials), both at baseline and Week 68 (Table 3). Additionally, IWQOL‐Lite‐CT composite scores were significantly higher for patients with weight loss of 5% or more compared with patients who experienced weight gain at Week 68 (*P* < 0.0001 for all composites in both trials).

### 3.6 Responsiveness

Table 3 summarizes evidence supporting the responsiveness, or ability to detect change, of the IWQOL‐Lite‐CT composites based on analysis of both STEP 1 and STEP 2 data. While the effect size estimates and standardized response means based on the data from STEP 1 are generally moderate in magnitude, those based on the data from STEP 2 are a bit smaller but still supportive of responsiveness. The paired *t* tests, comparing IWQOL‐Lite‐CT scores at baseline and Week 68, were statistically significant for all composites in both trials, providing further support for the ability of these scores to detect change.

### 3.7 Interpretation of change

Based on the primary anchor (1‐point improvement in PGI‐S score within STEP 1), IWQOL‐Lite‐CT responder thresholds are 14.6 points for the IWQOL‐Lite‐CT Physical Function composite score, 13.5 points for the IWQOL‐Lite‐CT Physical composite score, 16.2 points for the IWQOL‐Lite‐CT Psychosocial composite score, and 16.6 points for the IWQOL‐Lite‐CT Total score (Table 4). All supportive estimates, including those computed based on data from STEP 2, were somewhat smaller in magnitude (see Table 4). As such, the thresholds based on the primary anchor are recommended for use in future clinical studies to identify patients with meaningful responses to treatment.

**TABLE 4:** Interpretation of change from baseline to Week 68 (mean, median, *n*), transformed IWQOL‐Lite‐CT scores

Study/method	IWQOL‐Lite‐CT total	IWQOL‐Lite‐CT Physical	IWQOL‐Lite‐CT Physical Function	IWQOL‐Lite‐CT psychosocial
**STEP 1**				
**PGI‐S: 1‐point improvement**	**16.58, 13.8, 233**	**13.51, 10.7, 628**	**14.59, 15.0, 628**	**16.24, 14.4, 524**
PGI‐C: moderately better	12.20, 11.3, 208	10.62, 10.7, 351	11.41, 10.0, 351	15.14, 13.5, 394
5–10% weight loss	9.67, 8.8, 315	8.51, 7.1, 315	9.63, 10.0, 315	10.29, 9.6, 315
Half SD	10.54	11.57	12.07	11.46
SEM	5.91	8.02	9.10	6.64
**STEP 2**				
PGI‐S: 1‐point improvement	9.92, 8.8, 133	11.45, 10.7, 354	12.36, 10.0, 354	9.33, 7.7, 305
PGI‐C: moderately better	6.13, 5.0, 158	5.43, 3.6, 256	5.82, 5.0, 256	7.02, 5.8, 244
5–10% weight loss	7.06, 6.3, 272	7.17, 7.1, 272	7.79, 7.5, 272	7.00, 5.8, 272
Half SD	9.80	11.52	11.98	10.19
SEM	6.31	8.33	9.24	7.44

Abbreviations: IWQOL‐Lite‐CT, Impact of Weight on Quality of Life–Lite Clinical Trials Version; PGI‐C, patient global impression of change; PGI‐S, patient global impression of status; SEM, standard error of measurement.

## 4 DISCUSSION

The IWQOL‐Lite‐CT has been rigorously developed to assess weight‐related changes in physical and psychosocial functioning in patients with overweight and obesity and in accordance with FDA recommendations and guidance documents. While the development and psychometric evaluation processes are also consistent with recommendations provided by ISOQOL and the COnsensus‐based Standards for the selection of health Measurement INstruments (COSMIN) for use in research and clinical practice,^25^, ^32^, ^33^ FDA requirements for PRO measures used to support product labelling are more generally more stringent and specific,^21^, ^24^ particularly as they relate to documentation of input from the target patient population throughout the development process. The IWQOL‐Lite has been used in many trials for pharmacological treatments, and the IWQOL‐Lite‐CT offers the advantage of having been developed and validated specifically for use in a clinical trial setting. As such, it is expected to be more responsive to improvements in patient functioning within this context of use.

Importantly, both qualitative research and psychometric evaluations have been conducted in patients with overweight and obesity, both with and without T2D, to ensure and support the content validity and measurement properties of the IWQOL‐Lite‐CT within the broad spectrum of individuals typically seen in pharmacological trials for weight management. As such, the IWQOL‐Lite‐CT enables the evaluation, from the patient's perspective, of the impact and meaningfulness of weight loss in a clinical trial setting and can inform decision making regarding the impact of specific weight‐management treatments. In this study, confirmatory evaluations of the final, 20‐item IWQOL‐Lite‐CT were conducted with the use of data from STEP 1 and STEP 2, two phase 3a trials of subcutaneous semaglutide (2.4 mg) for weight management, supplementing and extending results pertaining to developmental versions of this measure.^23^ Taken together, the results of the initial and confirmatory psychometric evaluations of the IWQOL‐Lite‐CT strongly support the structure and scoring of this measure, as well as its reliability, validity, and ability to detect change in patients with overweight or obesity, including those with and without T2D. It should be noted that longitudinal correlations between the IWQOL‐Lite‐CT Psychosocial composite scores and SF‐36v2 MCS, MH, and Social Functioning scores were somewhat smaller than hypothesized in the STEP 2 analyses. We believe this was likely because only small changes in SF‐36v2 scores pertaining to psychosocial constructs were observed over the course of the trial.

The results of these analyses should be interpreted in the context of several strengths and limitations. A key strength of the study is its use of data from two large, multinational phase 3 trials. Nonetheless, the psychometric evidence supporting the IWQOL‐Lite‐CT has primarily been generated through analysis of data from pharmaceutical clinical trials for weight management. As such, it is unknown whether the results could be generalized to other contexts of use or influenced by the type of weight‐loss intervention.

## 5 CONCLUSIONS

The IWQOL‐Lite‐CT is appropriate for assessing weight‐related physical and psychosocial functioning in populations commonly targeted for weight management clinical trials. Given the strong support for each composite score, the selection of outcomes based on the IWQOL‐Lite‐CT can be tailored to the goals of the clinical trial sponsor and context of use. While the five‐item Physical Function composite has been developed for the purpose of supporting labelling in the US, the seven‐item Physical, 13‐item Psychosocial, and Total scores provide a more comprehensive assessment of treatment benefit. Providing further flexibility, electronic versions of the IWQOL‐Lite‐CT are available for licensed use on several different platforms, including web and mobile applications, and in 49 different languages with an additional 16 in process.

## CONFLICT OF INTEREST

This study was conducted under a research contract between Novo Nordisk and RTI Health Solutions and was funded by Novo Nordisk. Ronette L. Kolotkin is a consultant for and shareholder of Novo Nordisk and a consultant for RTI Health Solutions and receives royalties from Duke University for the IWQOL‐Lite‐CT. Sheri E. Fehnel, Valerie S. L. Williams, Nicole Williams, and Shanshan Qin are salaried employees of RTI Health Solutions. Henrik Hjorth Meincke and Lisa von Huth Smith are salaried employees of Novo Nordisk.

## Supporting information


**Table S1** STEP 1: Longitudinal CFA factor loadings (standard errors) and fitness indices using baseline (*n* = 1945), Week 20 (*n* = 1831), and Week 68 (*n* = 1761) data
**Table S2** STEP 2: Longitudinal CFA factor loadings (standard errors) and fitness indices using baseline (*n* = 1186), Week 20 (*n* = 1133), and Week 68 (*n* = 1111) data
