# Supplementary material for: Confirmatory psychometric evaluations of the Impact of Weight on Quality of Life–Lite Clinical Trials Version (IWQOL‐Lite‐CT)
Source: Clin Obes. 2021 Jul 22;11(5):e12477. doi: 10.1111/cob.12477 (PMC9285468; doi:10.1111/cob.12477)
Supplement: Supplementary file 1 — Table S1 STEP 1: Longitudinal CFA factor loadings (standard errors) and fitness indices using baseline (n = 1945), Week 20 (n = 1831), and Week 68 (n = 1761) data Table S2 STEP 2: Longitudinal CFA factor loadings (standard errors) and fitness indices using baseline (n = 1186), Week 20 (n = 1133), and Week 68 (n = 1111) data [file COB-11-0-s001.docx]

# Supporting Information

Table S1. STEP 1: Longitudinal CFA Factor Loadings (Standard Errors) and Fitness Indices Using Baseline (n = 1,945), Week 20 (n = 1,831), and Week 68 (n = 1,761) Data

| **IWQOL-Lite-CT Item** | **Longitudinal CFA** | | | **Longitudinal CFA With Modifications** | |
| --- | --- | --- | --- | --- | --- |
|  | **Physical** | **Psychosocial** | **Physical** | | **Psychosocial** |
| 1. Trouble bending over | 0.74 (0.01) |  | 0.75 (0.01)^b,d^ | |  |
| 2. Tired or winded | 0.71 (0.01) |  | 0.72 (0.01)^c^ | |  |
| 3. Unable to stand comfortably | 0.69 (0.01) |  | 0.70 (0.01) | |  |
| 4. Uncomfortable in small seats | 0.75 (0.01) |  | 0.77 (0.01) | |  |
| 5. Bodily pain | 0.69 (0.01) |  | 0.69 (0.01)^c^ | |  |
| 6. Self-conscious eating in social settings |  | 0.66 (0.01) |  | | 0.66 (0.01) |
| 7. Less confident |  | 0.87 (0.01) |  | | 0.88 (0.01) |
| 8. Feel judged by others |  | 0.83 (0.01) |  | | 0.84 (0.01) |
| 9. Frustrated shopping for clothes |  | 0.83 (0.01) |  | | 0.82 (0.01)^a^ |
| 10. Feel bad or upset about pictures of |  | 0.85 (0.01) |  | | 0.84 (0.01)^a^ |
| 11. Feel down or depressed about weight |  | 0.85 (0.01) |  | | 0.85 (0.01) |
| 12. Less interested in sexual activity |  | 0.71 (0.01) |  | | 0.71 (0.01) |
| 13. Avoid social gatherings |  | 0.80 (0.01) |  | | 0.81 (0.01) |
| 14. Less productive |  | 0.79 (0.01) |  | | 0.77 (0.01)^b,c^ |
| 15. Lack energy |  | 0.81 (0.01) |  | | 0.75 (0.01)^b,c^ |
| 16. Not physically active | 0.81 (0.01) |  | 0.79 (0.01)^c,d^ | |  |
| 17. Unable to walk far/quickly | 0.80 (0.01) |  | 0.79 (0.01)^b,c,d^ | |  |
| 18. Worried about health |  | 0.58 (0.01) |  | | 0.57 (0.01)^d^ |
| 19. Self-conscious about weight |  | 0.84 (0.01) |  | | 0.82 (0.01)^e^ |
| 20. Frustrated or upset about weight |  | 0.87 (0.01) |  | | 0.86 (0.01)^e^ |
| **RMSEA** | **0.059** | | | **0.053** | |
| **CFI/TLI** | **0.936/0.938** | | | **0.950/0.950** | |
| **SRMR** | **0.063** | | | **0.057** | |

CFA = confirmatory factor analysis; CFI = comparative fit index; IWQOL-Lite-CT = Impact of Weight on Quality of Life–Lite Clinical Trials Version; RMSEA = root mean square error of approximation; SRMR = standardized root mean square residual; TLI = Tucker-Lewis Index.

Note: In the first CFA, each item was allowed to load on only the factor with which it is grouped for scoring purposes. In the second CFA, item residuals were allowed to correlate between (a) Items 9 and 10; (b) Item 14 with Items 1, 15, and 17; (c) Item 15 with Items 14, 2, 5, 16, and 17; (d) Item 18 with Items 1, 16, and 17; and (e) Items 19 and 20.

Table S2. STEP 2: Longitudinal CFA Factor Loadings (Standard Errors) and Fitness Indices Using Baseline (n = 1,186), Week 20 (n = 1,133), and Week 68 (n = 1,111) Data

| **IWQOL-Lite-CT Item** | **Longitudinal CFA** | | **Longitudinal CFA With Modifications** | |
| --- | --- | --- | --- | --- |
|  | **Physical** | **Psychosocial** | **Physical** | **Psychosocial** |
| 1. Trouble bending over | 0.72 (0.01) | — | 0.73 (0.01) | — |
| 2. Tired or winded | 0.74 (0.01) | — | 0.74 (0.01) | — |
| 3. Unable to stand comfortably | 0.72 (0.02) | — | 0.73 (0.02) | — |
| 4. Uncomfortable in small seats | 0.76 (0.02) | — | 0.77 (0.02) | — |
| 5. Bodily pain | 0.70 (0.02) | — | 0.70 (0.02) | — |
| 6. Self-conscious eating in social settings | — | 0.68 (0.02) | — | 0.68 (0.01) |
| 7. Less confident | — | 0.86 (0.01) | — | 0.86 (0.01) |
| 8. Feel judged by others | — | 0.86 (0.01) | — | 0.85 (0.01) |
| 9. Frustrated shopping for clothes | — | 0.83 (0.01) | — | 0.82 (0.01)^a^ |
| 10. Feel bad or upset about pictures | — | 0.85 (0.01) | — | 0.84 (0.01)^a^ |
| 11. Feel down or depressed about weight | — | 0.86 (0.01) | — | 0.86 (0.01) |
| 12. Less interested in sexual activity | — | 0.68 (0.02) | — | 0.67 (0.02) |
| 13. Avoid social gatherings | — | 0.81 (0.01) | — | 0.81 (0.01) |
| 14. Less productive | — | 0.80 (0.01) | — | 0.78 (0.01)^b^ |
| 15. Lack energy | — | 0.84 (0.01) | — | 0.81 (0.01)^b^ |
| 16. Not physically active | 0.82 (0.01) | — | 0.80 (0.01)^b,c^ | — |
| 17. Unable to walk far/quickly | 0.79 (0.01) | — | 0.79 (0.01)^c^ | — |
| 18. Worried about health | — | 0.55 (0.02) | — | 0.53 (0.02)^c^ |
| 19. Self-conscious about weight | — | 0.81 (0.01) | — | 0.79 (0.01)^d^ |
| 20. Frustrated or upset about weight | — | 0.84 (0.01) | — | 0.82 (0.01)^d^ |
| **RMSEA** | **0.050** | | **0.047** | |
| **CFI/TLI** | **0.945/0.947** | | **0.952/0.953** | |
| **SRMR** | **0.057** | | **0.055** | |

CFA = confirmatory factor analysis; CFI = comparative fit index; IWQOL-Lite-CT = Impact of Weight on Quality of Life–Lite Clinical Trials Version; RMSEA = root mean square error of approximation; SRMR = standardized root mean square residual; TLI = Tucker-Lewis Index.

Note: In the first CFA, each item was allowed to load on only the factor with which it is grouped for scoring purposes. In the second CFA, item residuals were allowed to correlate between (a) Items 9 and 10, (b) Item 15 with Items 14 and 16, (c) Item 18 with Items 16 and 17, and (d) Items 19 and 20.
